# Chest wall pseudotumor: a case of non-tuberculous mycobacterial infection

**DOI:** 10.1186/s12879-021-05843-z

**Published:** 2021-02-19

**Authors:** Yutaka Shishido, Hiroshi Hamakawa, Kazuhiro Minami, Shigeo Hara, Yutaka Takahashi

**Affiliations:** 1 The Department of General Thoracic Surgery, Kobe City Medical Centre General Hospital, 2-2-1, Minatojimaminamimachi, Chuo-ku, Kobe, Hyogo 650-0047 Japan; 2 The Department of Diagnostic Pathology, Kobe City Medical Centre General Hospital, Kobe, Japan

**Keywords:** Non-Tuberculous mycobacteria, Chest wall, Pseudotumor, Case report

## Abstract

**Background:**

Non-tuberculous mycobacterial (NTM) infections are increasing worldwide, making them an international public health problem. Surgical management is often indicated for localized infectious disease; however, most surgeons are unaware of the potential risks of transmission during surgery.

**Case presentation:**

An 88-year-old Asian female was referred to our hospital for a tumor in the right lateral thoracic region. One month prior, she had a feeling of fullness and complained of localized pain and warmth in the right lateral thoracic wall. Pain and warmth gradually resolved without intervention; however, the fullness was getting worse. Computed tomography (CT) scan showed a mass of approximately 65 × 30 mm with an osteolytic change, involving the right 8th rib. Based on the rapid growth rate and CT findings, we strongly suspected a malignant chest wall tumor, and *en bloc* tumor resection with the 8th rib was performed. When the specimen was cut, a large amount of viscous pus was drained and its culture showed growth of *Mycobacterium avium*. Microscopically, the non-caseating epithelioid cell granuloma extended into the rib, infiltrating the bone cortex. On follow-up 1 month after discharge, there were no signs of infection or other adverse events associated with the surgery.

**Conclusions:**

Herein, we report about a patient with a mass diagnosed as an NTM abscess involving the rib cage, which was confused with a malignant tumor and eventually diagnosed following surgical excision. This report emphasizes the need to be aware of the possibility of NTM infection and take appropriate precautions if the patient has a rapidly growing mass in the chest wall.

## Background

The incidence and prevalence of non-tuberculous mycobacterial (NTM) infections are increasing worldwide, making it an international public health issue [1]. The main site of infection is the lung, but it can also occur in multiple organs. The diagnosis of NTM disease is challenging and requires discussion among clinicians, radiologists, and microbiologists [2]. Herein, we report a case of mass forming NTM abscess involving the rib cage, which was confused with a malignant tumor and eventually diagnosed following surgery.

## Case presentation

**Fig. 1:**
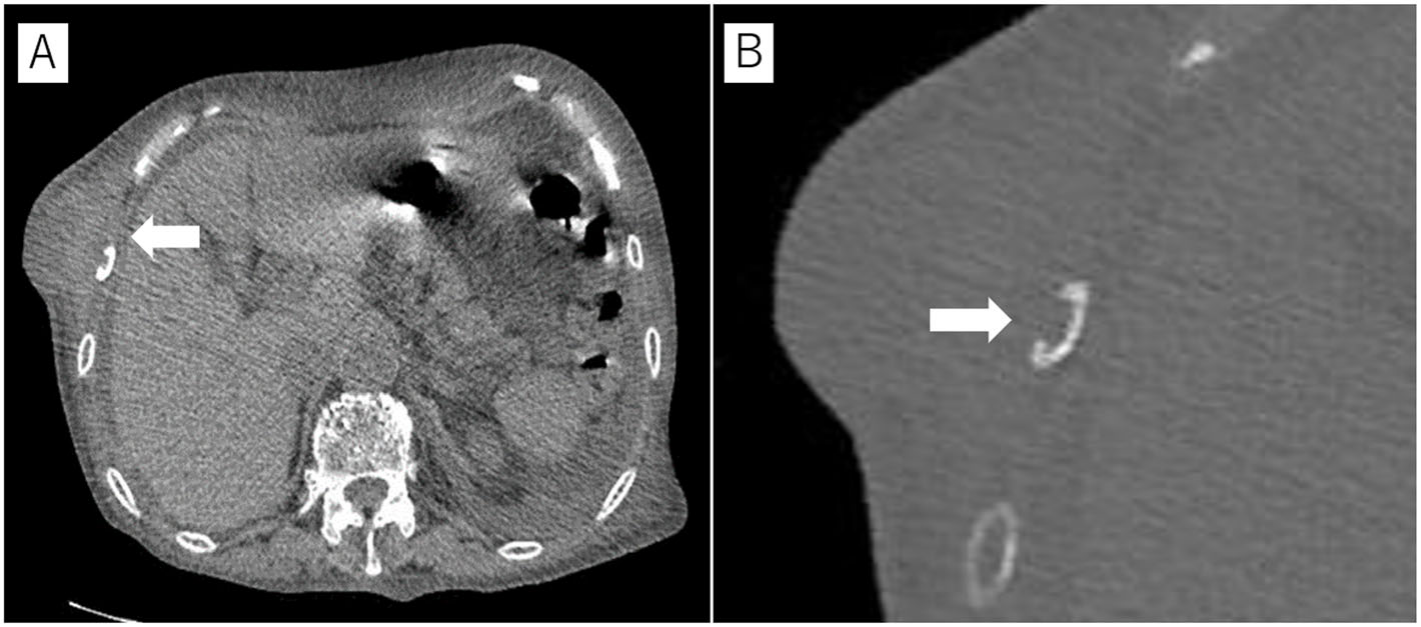
Computed tomography scan. **a** A mass in the chest wall, approximately 65 × 30 mm. **b** Right 8th rib with osteolytic change

**Fig. 2:**
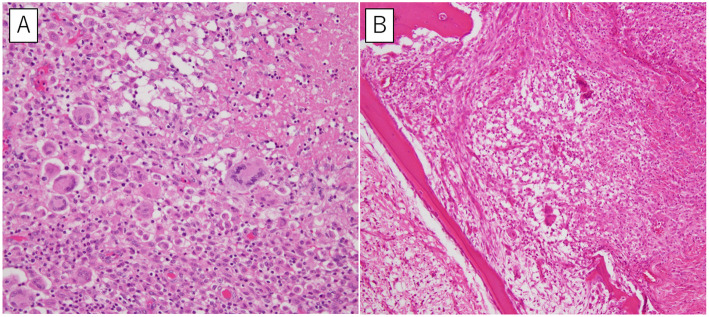
Hematoxylin and eosin staining (magnification 200×). **a** Non-caseating epithelioid cell granuloma with multinucleated giant cell formation. **b** Infiltration of granulomatous lesions into the bone cortex

An 88-year-old Asian female was referred to our hospital for evaluation and treatment of a tumor in the right lateral thoracic region. One month prior, she had a feeling of fullness and complained of localized pain and warmth in the right lateral thoracic wall. However, she did not present with fever, dyspnea, cough, hemoptysis, fatigue or weight loss. Pain and warmth gradually resolved without intervention; however, the fullness was getting worse. The doctor whom she visited previously examined the lesion by ultrasonography and estimated it as a soft tissue tumor. She was under medication for Alzheimer’s disease, hypertension, and chronic heart failure, and had a 25 pack-year smoking history but she had already quit smoking 3 years prior. And she did not have any immunosuppressive condition except the senility. On admission, she was afebrile with stable vital signs and had a lump in the right lateral thoracic region. The lump was non-tender, soft, and immobile. Lymphadenopathy or palpable lymph nodes were not detected. Laboratory examination showed white blood cell counts of 7200 μL and C-reactive protein level of 5.03 mg/dL. Computed tomography (CT) scan showed a mass of approximately 65 × 30 mm (Fig. 1a) with an osteolytic change, involving the right 8th rib (Fig. 1b), while there was no remarkable intrathoracic pulmonary lesion. Based on the rapid growth rate and CT findings, we strongly suspected a malignant chest wall tumor. Therefore, *en bloc* tumor resection with the 8th rib was performed. Macroscopically, the specimen contained a grayish-white solid mass 7.5 × 5.5 × 4.0-cm in size. When the specimen was cut, a large amount of viscous pus was drained. Microscopically, the specimen was characterized by a necrotic focus and non-caseating epithelioid cell granuloma with multinucleated giant cells (Fig. 2a). The granulomatous lesions extended into the rib, infiltrating the bone cortex (Fig. 2b). Ziehl-Neelsen staining of the pus smear revealed the presence of acid-fast bacilli, and its culture showed growth of *Mycobacterium avium*. Finally, the definite diagnosis was *M. avium* abscess involving the 8th rib. After the operation, she did not receive any antibiotic treatment for NTM because of her senility and completeness of anatomical resection of the lesion, and she was discharged uneventfully on postoperative day 12. On follow-up 1 month after discharge, there were no signs of infection or other adverse events associated with the surgery.

## Discussion and conclusions

NTM refers to mycobacteria other than *M. tuberculosi*s complex, *M. leprae*, and *M. ulcerans* [3]. NTM can cause a variety of medical problems, such as pulmonary disease, lymphadenitis, skin disease, and other extrapulmonary infections. Gray et al. reviewed about the 17 cases of NTM vertebral osteomyelitis in the absence of acquired immunodeficiency syndrome. In the report, 9 of the 17 had no known immunodeficiency. Furthermore, 4 of the 9 (24%) had isolated lesion, not disseminated lesions [4]. Thus, as far as we searched, although primary chest wall tuberculosis has been reported in different literatures, there is no report of an isolated NTM mass forming abscess with the osteolytic change in the chest wall, particularly in immunocompetent patients.

The route of infection in humans has not been completely understood, but the formation of aerosols that contain NTM arising from water, soil, and biofilm can be the sources of infection [5]. Surgical smoke, which is aerosol produced by surgical instruments, such as electrocautery scalpel, contains potentially harmful substances and transmission of infectious diseases might occur when bacterial particles are inhaled [6]. Although it is unknown whether surgical smoke could be a route of NTM transmission, surgeons and operating room personnel should take appropriate precautions to protect themselves from secondary infection.

Generally, for the diagnosis of NTM disease, it is essential to exclude other infectious disorders. Therefore, it is frequently challenging, and requires multidisciplinary discussion. For localized NTM infections of the skin and subcutaneous tissue, the diagnosis is based on the microbial culture of the drainage material or tissue biopsy [7]. In the present case, based on the rapid growth rate and CT findings, a malignant chest wall tumor was strongly suspected, and the *en bloc* tumor resection was performed before establishing the definite diagnosis. We should have suspected the possibility of infectious diseases and performed simple, urgent and less invasive procedures, such as needle biopsy, and more advanced imaging tests before surgery for rapid management of the case and to decrease the risk of transmission.

Treatment of NTM infection is difficult owing to the relative lack of susceptibility to currently available antibiotics and the difficulty in enduring a prolonged course of multiple drugs because of their adverse effects [7, 8]. Surgical management is often indicated in localized infectious disease, although the criteria for performing the surgery has not been clearly established [9]. In this case, the patient had no postoperative complications and there were no infectious events among those who were involved in this surgery. Complete surgical resection of the localized infectious lesions could be a viable option for patients who have the potential risk of multiple medications.

In conclusion, we report about a patient with a case of mass forming NTM abscess involving the rib cage, which was confused with a malignant tumor and eventually diagnosed following surgical excision. This report emphasizes the need to be aware of the possibility of NTM infection and take appropriate precautions if the patient has a growing mass in the chest wall.

## Data Availability

All data and materials of this article are available from the corresponding author.

## Abbreviations

NTM: Non-tuberculous mycobacteria
CT: Computed Tomography
